# Gender inequalities in prescribing and initiation patterns of guideline-recommended drugs after acute myocardial infarction

**DOI:** 10.1186/s12889-025-21396-1

**Published:** 2025-01-16

**Authors:** Irene López-Ferreruela, Sara Malo, Blanca Obón-Azuara, María José Rabanaque, Adriana Gamba, Sara Castel-Feced, Isabel Aguilar-Palacio

**Affiliations:** 1 Torreramona Health Centre, Primary Care, Servicio Aragonés de Salud (SALUD), Zaragoza, Spain; 2 Grupo de Investigación en Servicios Sanitarios de Aragón (GRISSA), Fundación Instituto de Investigación Sanitaria de Aragón (IIS Aragón), Zaragoza, Spain; 3 Department of Preventive Medicine and Public Health, University of Zaragoza, Zaragoza, Spain; 4 Research Network on Chronicity, Primary Care and Health Promotion (RICAPPS), Carlos III Health Institute (ISCIII), Madrid, Spain; 5 Intensive Medicine Service, Lozano Blesa Clinical University Hospital, Servicio Aragonés de Salud (SALUD), Zaragoza, Spain; 6 Department of Statistical Methods, University of Zaragoza, Zaragoza, Spain

**Keywords:** Myocardial infarction, Drugs prescription, Medication adherence, Secondary prevention, Gender inequalities, Real-world data

## Abstract

**Background:**

European guidelines recommend the prescription of certain drugs after acute myocardial infarction (AMI). The existence of gender differences in pharmacological treatment after an AMI has been described. This study aims to describe and analyse, using real-world data (RWD), whether there are gender differences in the prescribing patterns and initiation of treatment in secondary prevention after a first AMI, and which are the factors that explain these differences.

**Methods:**

A population-based observational study of RWD was conducted in the CARhES (CArdiovascular Risk factors for hEalth Services research) cohort. The study included subjects who had experienced a first episode of AMI between 2017 and 2022, had survived the event, and had a minimum follow-up of 180 days.

**Results:**

3,975 subjects were followed 180 days after a first AMI. Women (27.8% of the study population) were older and had more comorbidities. Of the main guideline-recommended drugs, antiplatelets, lipid modifying agents and beta-blockers, were prescribed less often in women. Comedications such as rivaroxaban and calcium channel blockers were more likely to be prescribed in women. The proportion of subjects initiating treatment was similar in both genders.

Overall, age and morbidity burden were the main contributors to differences in the prescribing patterns. Living in an urban area seemed to be a protective or mitigating factor. There were controversial results regarding socioeconomic level.

**Conclusion:**

In our study population, women are older, have greater comorbidities and lower socioeconomic status. Despite this, gender inequalities in the prescribing patterns after a first AMI remains, as women appear to experience less therapeutic effort. It is crucial to analyse them from an intersectional perspective, considering the influence of multiple axes of inequality on health, in order to develop gender-sensitive strategies with a multidisciplinary approach.

**Supplementary Information:**

The online version contains supplementary material available at 10.1186/s12889-025-21396-1.

## Background

Although significant progress has been made in prevention and treatment, cardiovascular disease (CVD) remains a major public health concern. It continues to hold its position as the leading cause of morbidity and mortality both in Europe and worldwide [1, 2]. In Europe, CVD represents the primary cause of death among men in all but twelve countries and remains the leading cause of death among women in all but two countries [3]. Worldwide, an estimated 17 million people die annually from CVD, mainly from stroke and acute myocardial infarction (AMI) [4, 5].

Within the disease process, health care following AMI (so-called secondary prevention) is essential to reduce mortality, increase survival and improve cardiac function, minimizing the occurrence of new events [6–8]. This secondary prevention involves a combination of elements including treatment prescription and initiation to cardioprotective drugs [9–11]. The European Society of Cardiology (ESC) recommends prescribing four main groups of drugs for secondary prevention after AMI: a combination of platelet aggregation inhibitors (aspirin and, in some cases, a P2Y12-inhibitor), a beta-blocker, lipid lowering therapy (statins and, in some cases, ezetimibe), and renin–angiotensin–aldosterone system inhibitors, including either an angiotensin-converting enzyme inhibitors (ACE-I), an angiotensin receptor blocker (ARB) or a mineralocorticoid receptor antagonist (MRA) [12–15].

The existence of gender differences in pharmacological treatment after an AMI has been described in the literature [16, 17]. These differences, in addition to the absence of gender-specific treatment differentiation or gender-specific clinical trials, result in a heightened vulnerability for women [6, 18–20].

However, research on gender inequalities in prescribing patterns of guideline-recommended long-term drugs following AMI remains limited, as do analyses of treatment initiation in patients prescribed these drugs [16]. Moreover, the use of Real World Data (RWD), defined as “data relating to patient health status and/or the delivery of healthcare, routinely collected from various sources,” provides valuable insights [21–23]. RWD enables the analysis of multiple variables from diverse information sources, offering a more comprehensive exploration of gender differences [21–24].

Therefore, the objective of this study is to describe and to analyse, using RWD, whether there are gender differences in prescribing and initiation patterns of guideline-recommended drugs in secondary prevention after a first episode of AMI, and which are the factors that explain these differences.

## Methods

### Study design, setting and population

Population-based observational study conducted in the CARhES (CArdiovascular Risk factors for hEalth Services research) cohort. This is a population dynamic open cohort, using RWD collected in the Aragón Health Service. It includes all people aged ≥ 16 years old with a diagnosis of hypertension, diabetes mellitus and/or dyslipidemia in the Spanish region of Aragón [25]. The study is based on data from the CARhES cohort. The CARhES cohort protocol was approved by the Ethics Committee for Clinical Research in Aragon (CEICA PI21/148). As there was no direct contact or interaction with the study participants and given the retrospective and population characteristics of the study, informed consent was waived by the Ethics Committee.

**Fig. 1:**
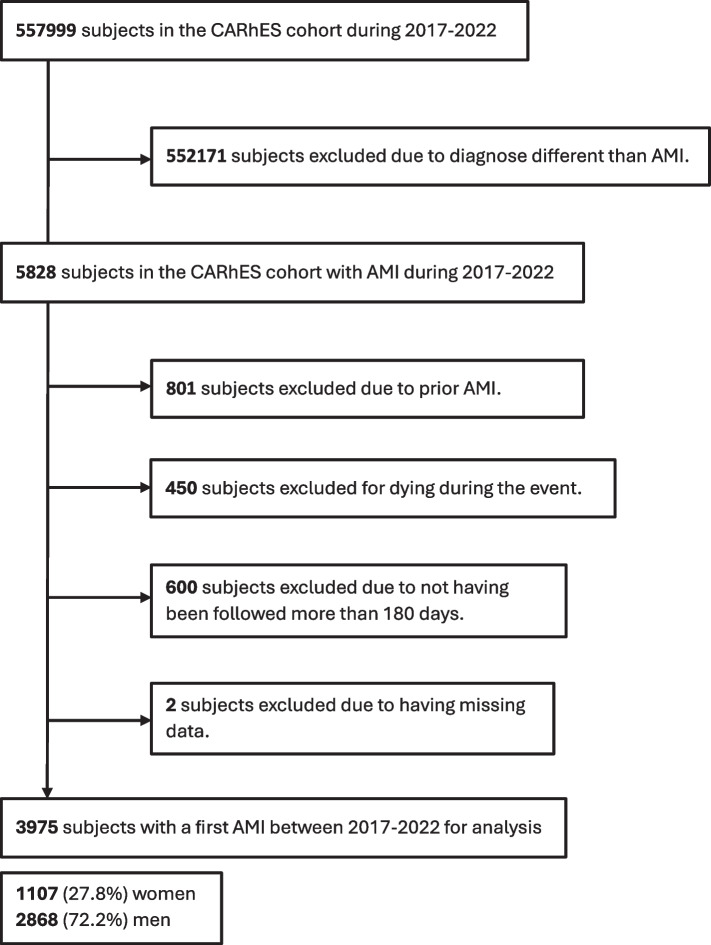
General outline of the study. Study flowchart of subjects’ inclusion and exclusion for the study. CARhES, CArdiovascular Risk factors for hEalth Services research. AMI, acute myocardial infarction

For the present study, subjects in the CARhES cohort with a first episode of AMI registered from 2017 to 2022 were included. We excluded subjects with a recorded diagnosis of AMI prior to the start of cohort follow-up, who died during the AMI, who were not followed up for more than 180 days, or for whom complete information was not available in the database. A flowchart of the study population selection criteria is presented in Fig. 1.

### Data sources and variables

Data were obtained from the CARhES cohort through BIGAN, a data lake that integrates the information systems of the Aragón Health Service. For the purposes of our study, we grouped the variables into different categories: sociodemographic, clinical and pharmacological treatment.

Regarding sociodemographic data, the following variables were registered at the time of the event: date of birth, gender, initial cardiovascular risk factor diagnosed, which is required for inclusion in the cohort (hypertension, diabetes mellitus and/or dyslipidemia), socioeconomic status, area of residence and institutionalization. Socioeconomic status was defined as different income categories: subjects with active employment with income below 18,000€ per year, active with income above 18,000€ per year, unemployed, pensioners with income below 18,000€ per year and free pharmacy, pensioners with income above 18,000€ per year and other status (including mutual, special conditions or uninsured subjects). Residential areas were differentiated between urban and rural areas, according to the basic healthcare area in which the subject resided.

In relation to clinical information, AMI was identified using code I21 of the International Classification of Diseases (ICD-10). Type of AMI was categorized by ICD-10 codes, differencing ST-elevation myocardial infarction (STEMI) as codes from I21.0 to I21.3, non-ST- elevation myocardial infarction (NSTEMI) as code I21.4 and other AMI as codes from I21.9 to I21.A. Morbidity adjusted groups (GMA) data is an information source that considers all medical diagnoses available in primary healthcare and hospital discharge records (Minimum Basic Data Set of Hospital Discharges). It provides different variables, such as: a summary of the main comorbidities presented in each subject, the number of chronic pathologies, the number of systems affected and their morbidity burden (obtained from the aggregation of the patient’s different diagnoses). The number of pathologies refers to the total number of different diagnosed diseases a patient has, as identified by the ICD system. The diagnosed diseases are grouped according to the organ system affected (e.g. cardiovascular, respiratory, endocrine). Finally, the morbidity burden quantifies the overall impact of the patient’s health conditions on their functional status and health care needs. It takes into account the severity of the disease, the patient’s comorbidities and the use of healthcare resources. More information on GMA can be found elsewhere [26].

With regard to pharmacological treatment, we selected those drugs recommended in the European guidelines for secondary prevention [12–15]: antiplatelets, beta-blockers, lipid modifying agents, ACEI/ARBs, and MRAs. We also considered some other comedication recommended, such as, new anticoagulants, nitrates, calcium channel blockers (CCBs) and proton pump inhibitors (PPIs). We identified these drugs using the anatomical therapeutic chemical (ATC)-code, version 2024 [27]. Antiplatelets included the drugs with ATC-code B01AC, beta-blockers the ATC-code C07 (all beta-blocking agents and combinations), lipid modifying agents the ATC-code C10, ACE-I/ARBs the ATC-code C09, MRAs the code C03DA (only considered in subjects with diabetes or heart failure), new anticoagulants the ATC-codes B01AF01 (rivaroxaban) and B01AE07 (dabigatran etexilate), nitrates the ATC-code C01DA, CCBs the ATC-code C08 (for those subjects without a C07 prescription or special cases) and PPIs the ATC-code A02.

In Aragón, chronic treatments are prescribed for a maximum of 12 months and can be renewed later by the practitioner. Once prescribed, the patient has 10 days to collect it from the pharmacy. There is no limit to the number of different medicines that can be collected at the same time and the patient can receive medicines for up to 3 months of treatment. Based on these criteria; to study the prescribing patterns, our population was classified into two groups: those new users who started a new treatment with the drug of interest within 30 days after the AMI (new users) and those who had an active prescription before the AMI and continued with the treatment after the AMI (former users).

The European-funded Ascertaining Barriers to Compliance (ABC) project proposed a new medication adherence taxonomy in 2012 to address the lack of consensus on terms and concepts related to medication adherence. Since its publication, the ABC taxonomy has been adopted by the International Society of Medication Adherence (ESPACOMP) and translated into several languages [28]. It divides adherence into three essential elements: initiation, implementation and discontinuation. Initiation represents the first stage of the process, when the patient takes the first dose of a prescribed medication [28].

In our study, to measure treatment initiation, we applied different criteria to assess treatment initiation in the two study groups: the new users and the former users. In new users, we checked for concordance between the date of treatment prescription and the date of dispensing, classifying as initiators those who had the drug dispensed within 30 days of the treatment prescription. In former users, those who had a dispensing within 30 days of the AMI were classified as initiators. We used a 30-day period because it is possible for former users to continue taking their previous medication after discharge from hospital and, once finished, to visit the doctor to renew the prescription. In these cases, the date used to estimate initiation would be the first, but the actual date would be the last.

### Statistical analyses

Sociodemographic and baseline clinical characteristics of the subjects studied were described using counts and proportions for categorical variables and means with confidence interval (CI) for continuous variables. Bivariate analyses between men’s and women’s characteristics were performed using Pearson’s Chi-squared test, and we compared means between groups using Student’s T-test.

Bivariate logistic regression analyses were performed to study gender differences in medication pattern of use (prescription and initiation) in the two previously defined groups (new users and former users). The threshold for statistical significance was set at *P* < 0.05.

Where we found significant gender differences, instead of the usual regression that measures only the differences, we used the Blinder-Oaxaca decomposition method to decompose these particular differences in health care attention and to analyse the factors that explained them. The Oaxaca decomposition method attempts to quantify how differences in the factors included in the analysis affect the observed differences.

This method decomposes the mean outcome observed differences between men and women into two components: the explained component, which captures the differences in the outcomes explained by the independent variables, and the unexplained component, which also captures all potential effects of unobserved variables [29]. A twofold decomposition applying Oaxaca R library and reference regression coefficients were calculated from a pooled regression model (https://cran.rproject.org/web/packages/oaxaca/vignettes/oaxaca.pdf).

All statistical analyses were performed using R 4.3.3. (R Core Team, 2022. A language and environment for statistical computing. R Foundation for Statistical Computing, Vienna, Austria. URL https://www.R-project.org/) [30] and JAMOVI (version 2.4) [Computer Software) Retrieved from https://www.jamovi.org [31].

## Results

A total of 3,975 subjects with a first AMI who were followed for at least 180 days conformed the study population. Of them, 1,107 (27.8%) were women and 2,868 (72.2%) men. The mean age at the time of the event was 69.4 years, which was significantly higher in women. The most prevalent type of AMI differed by gender, being non-ST elevation AMI more frequent in women and ST elevation AMI in men. Regarding socioeconomic status, pensioners < 18,000€ were the most represented in both men (42.40%) and women (67.60%). According to the place of residence, 2,807 (70.60%) of the population lived in urban areas and only 1.80% were institutionalized. Dyslipidemia was the most frequent cardiovascular risk factor in our population, and was slightly more prevalent among men, whereas hypertension was significantly more frequent in women. Diabetes was also slightly more prevalent among men, but the difference was not statistically significant. All the comorbidities studied, except chronic obstructive pulmonary disease and cirrhosis, were more prevalent in women. As for the health status, women had significantly a higher number of pathologies, affected systems and morbidity burden than men (*p* < 0.001) (Table 1).

**Table 1:** Comparison of socio-demographic and clinical characteristics for all the population studied and stratified by gender

	**Overall**		**Women**		**Men**		***p* values**
	**N**	**%**	**N**	**%**	**N**	**%**	
**Population**	3975	100	1107	27.8	2868	72.2	** < 0.001**
**Age at the event**^**a**^	69.4	69.0- 69.8	75.2	74.5–75.9	67.1	66.6–67.6	** < 0.001**
**AMI type**							
STEMI	2035	51.2	513	46.3	1522	53.1	** < 0.001**
NSTEMI	1847	46.5	558	50.4	1289	44.9	
Other AMI	93	2.3	36	3.3	57	2.0	
**Socioeconomic status**							
Employed < 18000€ per year	425	10.7	59	5.3	366	12.8	** < 0.001**
Employed ≥ 18000€ per year	430	10.8	41	3.7	389	13.6	
Unemployed	188	4.7	45	4.1	143	5.0	
Pensioners < 18000€ per year	1964	49.4	748	67.6	1216	42.4	
Pensioners ≥ 18000€ per year	825	20.8	172	15.5	653	22.8	
Other status	143	3.6	42	3.8	101	3.5	
**Residential area**							
Urban	2807	70.6	811	73.3	1996	69.6	**0.023**
Rural	1168	29.4	296	26.7	872	30.4	
**Institutionalized**	71	1.8	39	3.6	32	1.1	** < 0.001**
**Comorbidities**							
Hypertension	2776	69.8	879	79.4	1897	66.1	** < 0.001**
Dyslipidemia	3904	98.2	1071	96.7	2833	98.8	** < 0.001**
Diabetes Mellitus	1969	49.5	531	47.9	1438	50.1	0.220
Obesity	587	14.8	185	16.7	402	14.0	**0.032**
Heart failure	478	12.0	200	18.1	278	9.7	** < 0.001**
Chronic Obstructive Pulmonary Disease	392	9.9	71	6.4	321	11.2	** < 0.001**
Depression	622	15.6	288	26.0	334	11.7	** < 0.001**
Chronic Kidney Disease	902	22.7	289	26.1	613	21.4	** < 0.001**
Cirrhosis	141	3.5	39	3.5	102	3.6	0,959
Osteoporosis	312	7.8	278	25.1	34	1.2	** < 0.001**
Osteoarthritis	558	14.0	259	23.4	299	10.4	** < 0.001**
Dementia	126	3.2	75	6.8	51	1.8	** < 0.001**
**N chronic pathologies**^**a**^	6.51	6.4–6.6	7.55	7.4–7.7	6.1	6.0–6.2	** < 0.001**
**N affected systems**^**a**^	4.05	6.0–6.6	4.64	4.5–4.7	3.8	3.8–3.9	** < 0.001**
**Morbidity burden**^**a**^	12.3	12.1–12.5	14.1	13.7–14.5	11.6	11.4–11.8	** < 0.001**

N: number %: percentage

*AMI* acute myocardial infarction, *STEMI* ST elevation acute myocardial infarction, *NSTEMI* Non-ST elevation acute myocardial infarction

p: statistical significance *p* < 0.05, Pearson’s Chi-squared test, Student’s T-test

^a^Continuous variables were expressed as mean, confidence interval (CI) 95%

Gender differences in the prescribing patterns after a first AMI are shown in Table 2. For the main guideline-recommended drugs studied, men were significantly more likely than women to be prescribed the pharmacological groups of antiplatelets (90.7% vs. 88.1%), beta-blockers (74.3% vs. 70.3%) and lipid modifying agents (90.4% vs. 86.2%). On the other hand, women were more likely to be prescribed some of the comedications studied, such as CCBs (*p* = 0.003), or anticoagulant treatment with rivaroxaban (*p* = 0.047).

**Table 2:** Pharmacological treatment prescriptions by gender after a first AMI

	**Women (*n* = 1107)**		**Men (*n* = 2868)**		***p* values**
	**N**	**%**	**N**	**%**	
**Main guideline-recommended drugs**					
Antiplatelets	975	88.1	2600	90.7	**0.015**
Beta-blockers	778	70.3	2132	74.3	**0.010**
Lipid modifying agents	954	86.2	2593	90.4	** < 0.001**
ACE-I/ARBs	838	75.7	2138	74.5	0.452
MRA	84	7.6	176	6.1	0.097
**Comedications**					
Rivaroxaban	36	3.3	62	2.2	**0.047**
Dabigatran etexilate	9	0.8	28	1.0	0.631
Nitrates	473	42.7	1215	42.4	0.835
CCBs	170	15.4	338	11.8	**0.003**
PPIs	967	87.4	2497	87.1	0.807

N: number %: percentage, p: statistical significance *p* < 0.05, Pearson’s Chi-squared test

*ACE-I* angiotensin-converting enzyme inhibitors, *ARB* angiotensin receptor blocker, *MRA* mineralocorticoid receptor antagonist, *CCB* calcium channel blockers, *PPIs* proton pump inhibitors

A detailed analysis of separate prescribing patterns for new users and former users can be found in the Additional files online, Table 1.

Regarding treatment initiation (Table 3), the proportion of subjects initiating treatment was similar in both genders. However, women former users who were treated with PPIs seemed to have significantly higher frequency of initiating treatment (*p* = 0.016). No other significant gender differences in terms of initiation were found.

**Table 3:** Pharmacological treatment initiation after a first AMI according to the kind of prescription by gender

	**Initiation New users**					**Initiation Former users**				
	**Women**		**Men**		***p* values**	**Women**		**Men**		***p* values**
	**N**	**%**	**N**	**%**		**N**	**%**	**N**	**%**	
**Main guideline-recommended drugs**										
Antiplatelets	869	98.9	2332	99.2	0.377	51	53.1	119	47.8	0.375
Beta-blockers	582	96.8	1673	97.5	0.391	113	63.8	242	58.2	0.198
Lipid modifying agents	639	97.2	2035	98.1	0.169	147	61.8	285	55.0	0.082
ACE-I/ARBs	446	96.7	1299	97.4	0.479	192	50.9	398	49.5	0.648
MRA	61	96.8	137	97.9	0.661	4	19.0	14	38.9	0.208
**Comedications**										
Rivaroxaban	18	100.0	32	97.0	0.456	8	44.4	16	55.2	0.475
Dabigatran etexilate	5	100.0	12	100.0	-	4	100.0	7	43.8	0.144
Nitrates	341	91.9	857	89.6	0.210	46	45.1	96	37.1	0.160
CCBs	72	94.7	148	94.9	0.965	28	29.8	40	22.0	0.154
PPIs	491	96.8	1478	96.9	0.934	345	75.0	669	68.8	**0.016**

N: number %: percentage, p: statistical significance *p* < 0.05, Pearson’s Chi-squared test, Fisher’s Exact Test

*ACE-I* angiotensin-converting enzyme inhibitors, *ARB* angiotensin receptor blocker, *MRA* mineralocorticoid receptor antagonist, *CCB* calcium channel blockers, *PPIs* proton pump inhibitors

We conducted univariate models to determine gender differences in the prescribing patterns and initiation of pharmacological treatment. Data can be found in the Additional files online, Tables 2 and 3.

**Fig. 2:**
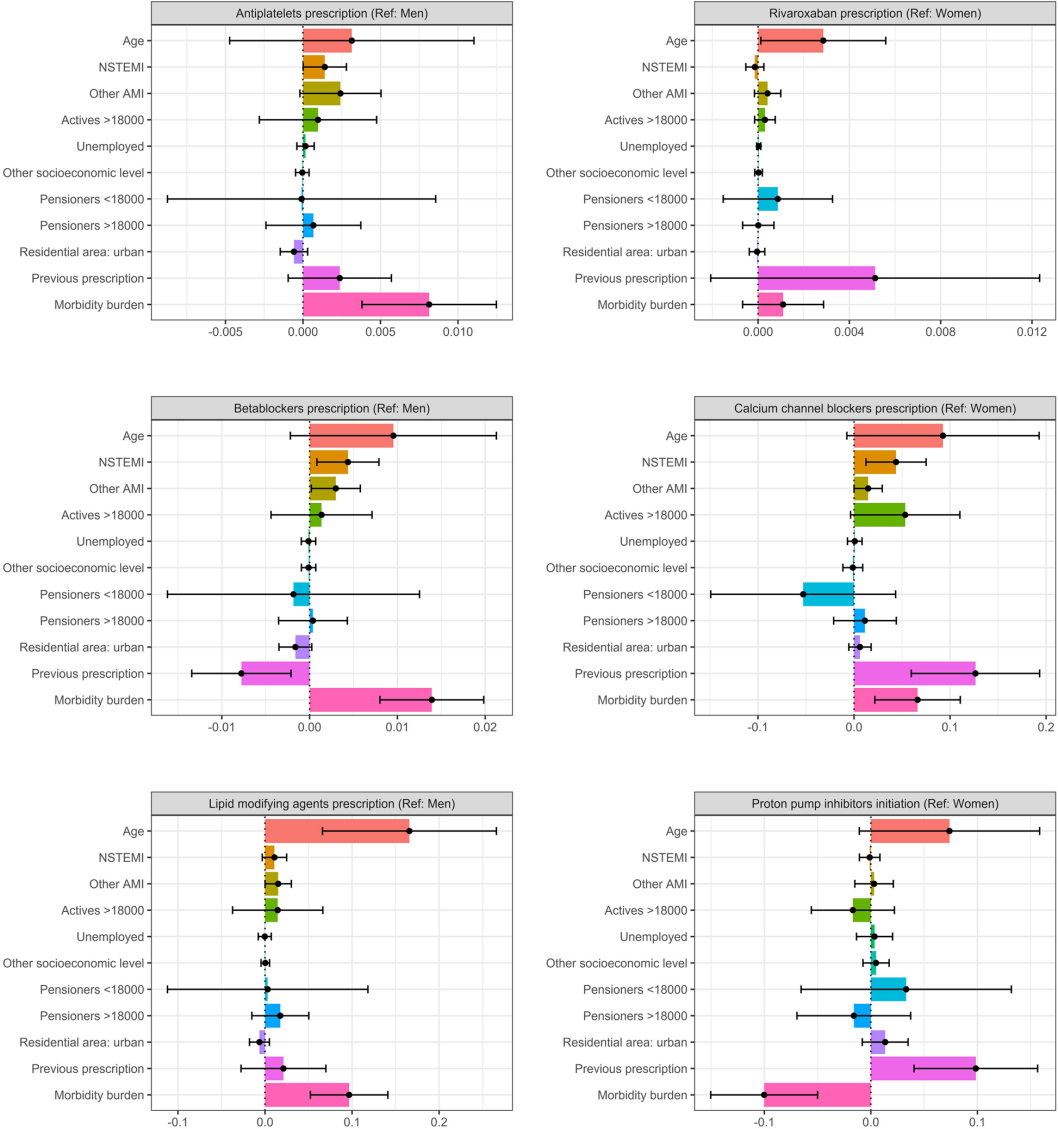
Decomposition of gender differences in treatment prescribing and initiation patterns. Oaxaca decomposition analyses. Ref; reference (gender taken as reference category); NSTEMI: Non-ST elevation acute myocardial infarction; AMI: acute myocardial infarction

In order to explain the gender differences observed in the prescribing patterns of some pharmacological groups, Oaxaca decomposition analyses were performed (Fig. 2). The explanatory variables included in the analyses were age, type of AMI, socioeconomic status, area of residence (urban or rural), previous prescription of the drug, and morbidity burden. The explained fraction of the observed gender differences was above 50% for all pharmacological groups studied, except for initiation of PPIs in former users (23.17%). This means that more than 50% of the observed gender differences are explained by the effect of these variables. The pharmacological group with the highest explained fraction was anticoagulant treatment with rivaroxaban in favour of women (96.82%), followed by antiplatelet drugs in favour of men (71.98%).

As shown in Fig. 2, age and morbidity burden were the most common variables that increased gender differences in the prescribing patterns of the main guideline-recommended drugs (antiplatelets, beta-blockers, and lipid-modifying agents) as well as certain co-medications (rivaroxaban and CCBs). This suggests that women were less likely to receive these drugs if they were older or had a higher burden of comorbidities.

The type of AMI was also a significant variable, particularly for antiplatelets and beta-blockers. Residing in an urban area appeared to attenuate gender disparities in the prescribing patterns for antiplatelets, beta-blockers, and lipid-modifying agents, with women in urban areas being likely to be prescribed these medications compared to those in rural areas.

For CCBs and anticoagulant treatment with rivaroxaban, both of which were more commonly prescribed to women, the existence of a previous prescription was associated with widening gender differences in favour of women, meaning that a previous prescription increased the likelihood of continuing treatment (Fig. 2).

Socioeconomic status, however, was controversial. Oaxaca analyses (Fig. 2) indicate that being a pensioner with an income below 18,000€ increased gender differences in the prescribing of rivaroxaban, with lower income associated with reduced prescribing rates for men. On the other hand, for CCBs, being a pensioner with an income below 18,000€ seemed to reduce gender differences, as prescription rates for men increased and approached those of women.

Similarly, in the case of beta-blockers, gender differences favouring women were reduced, as being a pensioner with an income below 18,000€ and having a previous prescription for the treatment increased the likelihood of a woman continuing treatment or being newly prescribed it (Fig. 2).

Regarding the initiation of PPIs by former users, our analysis (Fig. 2) suggests that having a previous prescription, older age, and being a pensioner with an income below 18,000€ increased gender differences in favour of women. In other words, men who were former users, were less likely to initiate PPIs due to their income, age, or previous prescription history. In contrast, having an income above 18,000€ and a higher morbidity burden seemed to reduce these differences and increase the likelihood of men initiating the drug.

## Discussion

The present study offers a comprehensive analysis of the existing gender differences in the prescribing and initiation patterns of guideline-recommended drugs following a first AMI. Our population appears to exhibit different sociodemographic and clinical characteristics according to gender. Women are generally older than men, have more comorbidities and a higher morbidity burden. After a first AMI, of the main guideline-recommended drugs, antiplatelets, lipid modifying agents and beta-blockers, were prescribed more often in men than in women. However, women were more likely to be prescribed comedications such as the anticoagulant rivaroxaban and CCBs. In terms of initiation, the proportion of subjects initiating the drugs studied was similar in both gender groups, with the only exception of former PPIs users.

Overall, the older age of women and their higher frequency of comorbidities are the main contributors to differences in prescribing, while living in an urban area seems to be a protective or mitigating factor for these differences. Regarding socioeconomic level, it would appear that is a factor which helps to reduce gender differences in relation to some drugs. However, for other drugs, it has the opposite effect, widening these differences.

### Possible explanations

In terms of sociodemographic characteristics, and in line with the existing literature, the proportion of men and women in our study is not balanced (27.8% of women). It has been observed that women with a first AMI are older and have a higher prevalence of risk factors and comorbidities than men [32–35]. As described by Madika et al. [36], the onset of CVD is later in women than in men, with an average delay of approximately 10 years. These differences may be due, among other factors, to hormonal status. However, the prevalence of cardiovascular risk factors increases with age, and the incidence of the disease tends to level off after the age of 65, especially in postmenopausal women [36].

In fact, the severity and lethality of the disease remain higher in women because of their more unfavourable risk profile, with a greater prevalence of risk factors and comorbidities [32–36].

In line with our recent scoping review [16], our findings on prescribing patterns of pharmacological treatments are similar to other studies and also similar to those obtained in other cardiovascular diseases [37, 38]. Women were less likely to receive guideline-recommended drugs such as antiplatelets, beta-blockers and lipid modifying agents. These differences are described in the literature [16, 34, 39–41]. Women are subject to less therapeutic effort and receive more obsolete and less current treatments than those recommended by clinical guidelines [16, 34, 39–41]. Even when prescribed, women are more likely to receive less intensive treatment and in lower doses [42–44]. The reasons for this disparity remain understudied [36, 42, 43]. However, some authors have suggested several factors that may contribute to this gender disparity for all the pharmacological groups studied, including an older age, a higher prevalence of comorbidities, a greater risk of exacerbations [45, 46], or an underestimation of CVD risk in women [44]. Sotorra-Figueroa et al. [34], supported by other studies [42–46], found a strong association between insufficient guideline-recommended drugs prescription and being an older woman. In addition, the absence of evidence-based guidelines specific for women [45, 46], the lack of trials with gender-specific analyses, or the historical underrepresentation of women in clinical trials [46, 47], may serve to exacerbate these gender differences, influencing treatment decisions and preventing equal effectiveness for women and men [44]. As a result, it is unclear whether guideline recommendations should be the same or not.

Furthermore, there is a lack of published literature that specifically addresses the reasons for gender inequalities in the prescribing patterns of specific treatments. Regarding the undertreatment of women with antiplatelets, Ruiz-Nodar et al. [32] suggested that the higher rates of anemia in women, their increased risk of bleeding complications, and higher mortality rates, may have led to lower prescribing patterns or to the prescription of more conservative antiplatelet therapies. In accordance with the above, the increased prescribing patterns of comedications in women, such as the new anticoagulants or PPIs, is consistent with this increased risk of bleeding, as well as the higher prevalence of other comorbidities with a haemorrhagic etiology [48, 49]. Other studies [34, 41, 44] reviewing statin therapies suggest that lower prescribing patterns in women could be due to a lower risk perception of the disease by the practitioner, lower perceived benefits of treatment, as well as real or perceived side effects commonly reported by women on this treatment. For beta-blockers, other studies found smaller differences in the prescribing patterns. This may be explained by the more frequent use of comedication in women for blood pressure control in relation to the presence of other comorbidities. Some trials suggested that there might be gender differences in the effectiveness of B-blockers [34, 50].

These gender differences highlight the necessity of the implementation of gender-specific clinical trials or, at least, balanced inclusion in clinical studies, gender-specific testing and guidelines, and the guarantee of equal access to healthcare for women undergoing cardiovascular treatment.

Consistent with existing evidence [34, 44–46], our study identified the morbidity burden and age as the main contributors to the observed gender differences in prescribing patterns, thereby reducing the likelihood of guideline-recommended drugs being prescribed to women, which suggests a lower therapeutic effort [34, 39–41]. Indeed, previous treatment prescription was identified as a significant factor increasing the likelihood of prescription for women, or continuation with prescribed treatment, even if these treatments are obsolete or not recommended by the guidelines.

In addition, literature has identified a number of socioeconomic factors being associated with risk and management of CVD. These include area of residence, socioeconomic status, and educational level [51]. Our findings showed that living in an urban area was a moderating factor for differences in prescribing. On the other hand, rural residence has been associated with poorer health control, as it increases barriers to health care access due to greater geographical dispersion or a paucity of healthcare professionals, particularly for women who tend to be older, institutionalised, have driving limitations or prioritize family and caring roles over their own health [51, 52].

The results of our study regarding socioeconomic level are controversial. However, higher socioeconomic levels have been found to narrow the gender disparities in the management of CVD [44, 53–55]. Unfortunately, women in Spain have a lower socioeconomic status due to the gender pay gap, which is particularly pronounced in retirement, potentially leading to an increase in these differences [56].

The reasons for the persistence of this gender bias in the prescribing patterns of treatment after acute myocardial infarction remain unclear [57, 58]. Several factors, including sociodemographic, disease-related, risk factor-related and others, seem to play an important role. It should be emphasized that AMI is a multifactorial condition, with risk factors often coexisting and modulated differently by gender. In this context, the relative impact of an individual risk factor is rather complex to assess, as is the impact of gender on management in clinical practice. The coexistence of comorbidities, female gender, ageism and in some cases, lower socioeconomic status, seems to increase treatment inequalities. The continued undertreatment of women following an AMI suggests a lack of adherence by health professionals to international guidelines on secondary prevention. Furthermore, the majority of studies have not addressed this issue from a gender perspective. These differences indicate a situation of gender inequality that requires further studies and analyses from an intersectional perspective to confirm this fact.

### Strengths and limitations

The main strength of our study is the use of BIGAN, the RWD lake, as the main source of valuable and rich data, integrating the information system of the Aragón Health Service. Thus, our study shows a high internal validity in the representativeness of the study population at an autonomic level. Another highlight is the use of the Blinder-Oaxaca decomposition method to analyse the particular differences in the prescribing and initiation patterns of treatment, which captures the differences in outcomes explained by the different variables and is also able to show potential effects of unobserved variables.

However, there are limitations to be considered. One limitation of working with registered diagnoses would be the impossibility of measuring the diagnostic bias of CVD. In other words, in our study we analysed the differences in people who had been diagnosed, leaving out those who had not been diagnosed. According to the literature, this situation is more common in women, who are often under-represented in studies because of a possible under-diagnosis of the pathology, possibly due to non-description of their symptoms in previous guidelines. Another possible limitation is that we do not have the reason why patients did not receive the specific drug, we only have prescription data. So, we cannot legitimize reasons for non-prescription by clinicians. We also have to consider a possible bias in the treatment initiation analyses due to the difficulty of measuring it in those subjects who had previously been prescribed the drug (former users), which we explain in detail in the methodology. Finally, we only analysed the prescription pattern and the initial collection of the drug. For future work, it will be of interest to also analyse factors associated with persistence, changes in treatment and its effect on secondary prevention.

## Conclusions

In essence, the results in our study population show that women are older, have more cardiovascular risk factors, and have greater morbidity. Women are also more likely to have lower socioeconomic status, particularly among pensioners, and are more frequently institutionalised.

Despite these factors, men are still more likely to be treated with guideline-recommended drugs. This suggests that healthcare professionals’ adherence to guideline recommendations for secondary prevention may be inadequate, as women appear to experience less therapeutic effort, particularly when they are older and have a higher morbidity burden. All these differences, combined with socioeconomic factors and place of residence, highlight the existence of gender inequalities in the treatment of AMI.

It is therefore crucial to address these inequalities in order to improve cardiovascular health worldwide. This requires an intersectional perspective analysis that considers the simultaneous and interacting effects of multiple axes of inequality, such as, socioeconomic status, gender, place of origin, or ageism, in order to develop gender-sensitive strategies with a multidisciplinary approach to achieve equitable health outcomes.

## Supplementary Information

Supplementary Material 1: Table 1. Pharmacological treatment prescribing patterns by gender and group of prescription after a first AMI.

Supplementary Material 2: Table 2. Bivariate regression. Pharmacological treatment prescription by group of prescription after a first AMI adjusted by gender.

Supplementary Material 3: Table 3. Bivariate regression. Pharmacological treatment initiation after a first AMI according to the kind of prescription adjusted by gender.

## Data Availability

All data generated or analysed during this study are included in this published article and its supplementary information files.

## Abbreviations

AMI: Acute myocardial infarction
RWD: Real World Data
CARhES: CArdiovascular Risk factors for hEalth Services research
CVD: Cardiovascular disease
ESC: European Society of Cardiology
ACE-I: Angiotensin-converting enzyme inhibitors
ARBs: Angiotensin receptor blockers
MRA: Mineralocorticoid receptor antagonist
CCB: Calcium channel blockers
PPIs: Proton pump inhibitors
CEICA: Clinical Research Ethics Committee of Aragón
ICD: International Classification of Diseases
STEMI: ST-elevation myocardial infarction
NSTEMI: Non-ST- elevation myocardial infarction
GMA: Morbidity adjusted groups
ATC: Anatomical therapeutic chemical
CI: Confidence Interval
N: Number
%: Percentage
P: Statistical significance
