# Supplementary material for: Gender inequalities in prescribing and initiation patterns of guideline-recommended drugs after acute myocardial infarction
Source: BMC Public Health. 2025 Jan 16;25:185. doi: 10.1186/s12889-025-21396-1 (PMC11740473; doi:10.1186/s12889-025-21396-1)
Supplement: Supplementary file 1 — Supplementary Material 1: Table 1. Pharmacological treatment prescribing patterns by gender and group of prescription after a first AMI. [file 12889_2025_21396_MOESM1_ESM.docx]

**ADDITIONAL FILE 1.**

**Table 1**. **Pharmacological treatment prescribing patterns by gender and group of prescription after a first AMI.**

|  | **New users** | | | | | **Former users** | | | | |
| --- | --- | --- | --- | --- | --- | --- | --- | --- | --- | --- |
|  | **Women (n= 1107)** | | **Men (n= 2868)** | | **p values** | **Women (n= 1107)** | | **Men (n= 2868)** | | **p values** |
|  | **N** | **%** | **N** | **%** |  | **N** | **%** | **N** | **%** |  |
| **Main guideline-recommended drugs** |  |  |  |  |  |  |  |  |  |  |
| Antiplatelets | 879 | 79 | 2351 | 82 | 0,063 | 96 | 9 | 249 | 9 | 0,992 |
| Beta-blockers | 601 | 54 | 1716 | 60 | **<0,001** | 177 | 16 | 416 | 15 | 0,239 |
| Lipid modifying agents | 716 | 65 | 2075 | 72 | **<0,001** | 238 | 21 | 518 | 18 | **0,013** |
| ACE-I/ARBs | 461 | 42 | 1334 | 47 | **0,006** | 377 | 34 | 804 | 28 | **<0,001** |
| MRA | 63 | 6 | 140 | 5 | 0,299 | 21 | 2 | 36 | 1 | 0,127 |
| **Comedications** |  |  |  |  |  |  |  |  |  |  |
| Rivaroxaban | 18 | 2 | 33 | 1 | 0,233 | 18 | 2 | 29 | 1 | 0,108 |
| Dabigatran etexilate | 5 | 0,5 | 12 | 0,4 | 0,886 | 4 | 0 | 16 | 1 | 0,432 |
| Nitrates | 371 | 34 | 956 | 33 | 0,914 | 102 | 9 | 259 | 9 | 0,857 |
| CCBs | 76 | 7 | 156 | 5 | 0,086 | 94 | 8 | 182 | 6 | **0,017** |
| PPIs | 507 | 46 | 1525 | 53 | **<0,001** | 460 | 42 | 972 | 34 | **<0,001** |

N: number %: percentage. p: statistical significance p<0.05

ACE-I: angiotensin-converting enzyme inhibitors; ARB: angiotensin receptor blocker.

MRA: mineralocorticoid receptor antagonist; CCB: calcium channel blockers, PPIs: proton pump inhibitors.

New users: population who started a new treatment with the drug of interest within 30 days after AMI.

Former users: population who had an active prescription before the AMI and continued with the treatment after.
