# Supplementary material for: Gender inequalities in prescribing and initiation patterns of guideline-recommended drugs after acute myocardial infarction
Source: BMC Public Health. 2025 Jan 16;25:185. doi: 10.1186/s12889-025-21396-1 (PMC11740473; doi:10.1186/s12889-025-21396-1)
Supplement: Supplementary file 2 — Supplementary Material 2: Table 2. Bivariate regression. Pharmacological treatment prescription by group of prescription after a first AMI adjusted by gender. [file 12889_2025_21396_MOESM2_ESM.docx]

**ADDITIONAL FILE 2.**

**Table 2**. **Pharmacological treatment prescription by group of prescription after a first AMI. Odds Ratios from gender comparison.**

|  | **New users** | | | **Former users** | | | **Overall population** | | |
| --- | --- | --- | --- | --- | --- | --- | --- | --- | --- |
|  | **Odds Ratio** | **CI (95%)** | **p values** | **Odds Ratio** | **CI (95%)** | **p values** | **Odds Ratio** | **CI (95%)** | **p values** |
| **Main guideline-recommended drugs** |  |  |  |  |  |  |  |  |  |
| Antiplatelets | 0.85 | 0.71 – 1.01 | 0.063 | 1.00 | 0.78 – 1.27 | 0.992 | 0.76 | 0.61 – 0.95 | **0.015** |
| Beta-blockers | 0.80 | 0.69 – 0.92 | **0.002** | 1.12 | 0.92 – 1.36 | 0.239 | 0.82 | 0.70 – 0.95 | **0.010** |
| Lipid modifying agents | 0.70 | 0.60 – 0.81 | **<0.001** | 1.24 | 1.04 – 1.47 | **0.013** | 0.66 | 0.54 – 0.82 | **<0.001** |
| ACE-I/ARBs | 0.82 | 0.71 – 0.94 | **0.006** | 1.33 | 1.14 – 1.54 | **<0.001** | 1.06 | 0.91 – 1.25 | 0.452 |
| MRA | 1.18 | 0.86 – 1.59 | 0.299 | 1.52 | 0.87 – 2.59 | 0.130 | 1.26 | 0.96 – 1.64 | 0.097 |
| **Comedications** |  |  |  |  |  |  |  |  |  |
| Rivaroxaban | 1.42 | 0.78 – 2.50 | 0.235 | 1.62 | 0.88 – 2.90 | 0.111 | 1.52 | 0.99 – 2.29 | **0.047** |
| Dabigatran etexilate | 1.08 | 0.34 – 2.92 | 0.885 | 0.65 | 0.18 – 1.77 | 0.436 | 0.83 | 0.37 – 1.70 | 0.631 |
| Nitrates | 1.01 | 0.87 – 1.17 | 0.914 | 1.02 | 0.80 – 1.30 | 0.857 | 1.02 | 0.88 – 1.17 | 0.835 |
| CCBs | 1.28 | 0.96 – 1.70 | 0.086 | 1.37 | 1.05 – 1.77 | **0.017** | 1.36 | 1.11 – 1.65 | **0.003** |
| PPIs | 0.74 | 0.65 – 0.86 | **<0.001** | 1.39 | 1.20 – 1.60 | **<0.001** | 1.03 | 0.84 – 1.27 | 0.807 |

CI: Confidence interval 95%. p: statistical significance p<0.05

ACE-I: angiotensin-converting enzyme inhibitors; ARB: angiotensin receptor blocker.

MRA: mineralocorticoid receptor antagonist; CCB: calcium channel blockers, PPIs: proton pump inhibitors.

New users: population who started a new treatment with the drug of interest within 30 days after AMI.

Former users: population who had an active prescription before the AMI and continued with the treatment after.
