# Supplementary material for: Gender inequalities in prescribing and initiation patterns of guideline-recommended drugs after acute myocardial infarction
Source: BMC Public Health. 2025 Jan 16;25:185. doi: 10.1186/s12889-025-21396-1 (PMC11740473; doi:10.1186/s12889-025-21396-1)
Supplement: Supplementary file 3 — Supplementary Material 3: Table 3. Bivariate regression. Pharmacological treatment initiation after a first AMI according to the kind of prescription adjusted by gender. [file 12889_2025_21396_MOESM3_ESM.docx]

**ADDITIONAL FILE 3.**

**Table 3**. **Pharmacological treatment initiation after a first AMI according to the kind of prescription. Odds Ratios from gender comparison.**

|  | **Initiation New users** | | | | **Former users** | | | |
| --- | --- | --- | --- | --- | --- | --- | --- | --- |
|  | **Odds Ratio** | **CI (95%)** | **p values** | **N** | **Odds Ratio** | **CI (95%)** | **p values** | **N** |
| **Main guideline-recommended drugs** |  |  |  |  |  |  |  |  |
| Antiplatelets | 0.71 | 0.33 – 1.59 | 0.379 | 3230 | 1.24 | 0.77 – 1.99 | 0.375 | 345 |
| Beta-blockers | 0.79 | 0.46 – 1.39 | 0.392 | 2317 | 1.27 | 0.88 – 1.83 | 0.198 | 593 |
| Lipid modifying agents | 0.68 | 0.40 – 1.20 | 0.171 | 2791 | 1.32 | 0.97 – 1.81 | 0.082 | 756 |
| ACE-I/ARBs | 0.80 | 0.44 – 1.52 | 0.479 | 1795 | 1.06 | 0.83 – 1.35 | 0.648 | 1181 |
| MRA | 0.67 | 0.11 – 5.17 | 0.663 | 203 | 0.37 | 0.09 – 1.25 | 0.127 | 57 |
| **Comedications** |  |  |  |  |  |  |  |  |
| Rivaroxaban | 26704232.14 | 0.00 – NA | 0.997 | 51 | 0.65 | 0.19 – 2.11 | 0.475 | 47 |
| Dabigatran etexilate | 1.00 | 0.00 – Inf | 1.000 | 17 | 148691304.95 | 0.00 – NA | 0.995 | 20 |
| Nitrates | 1.31 | 0.87 – 2.04 | 0.212 | 1327 | 1.39 | 0.88 – 2.22 | 0.160 | 361 |
| CCBs | 0.97 | 0.30 – 3.74 | 0.965 | 232 | 1.51 | 0.85 – 2.64 | 0.155 | 276 |
| PPIs | 0.98 | 0.56 – 1.79 | 0.934 | 2032 | 1.36 | 1.06 – 1.75 | **0.016** | 1432 |

CI: Confidence interval 95%. p: statistical significance p<0.05 N: number.

ACE-I: angiotensin-converting enzyme inhibitors; ARB: angiotensin receptor blocker.

MRA: mineralocorticoid receptor antagonist; CCB: calcium channel blockers, PPIs: proton pump inhibitors.

New users: population who started a new treatment with the drug of interest within 30 days after AMI.

Former users: population who had an active prescription before the AMI and continued with the treatment after.
